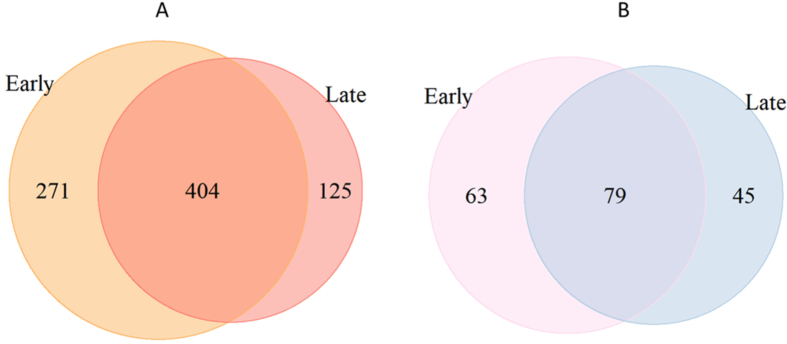# Exploring the molecular landscape of NNK-induced transformation: A comprehensive genome-wide CRISPR/Cas9 screening

**DOI:** 10.1016/j.gendis.2023.101131

**Published:** 2023-09-29

**Authors:** Trang Dinh, Mira Rahm, Zhenghe Wang, Christopher McFarland, Athar Khalil

**Affiliations:** Department of Genetics and Genome Sciences, Case Western Reserve University, Cleveland 44106, USA

Lung cancer is the leading cause of cancer-related deaths worldwide, with an estimated 1.8 million deaths in 2020 alone.^1^ While several risk factors are associated with lung cancer, smoking remains the most significant environmental cause of the disease.^1^ One of the most potent carcinogens in tobacco smoke is 4-(methylnitrosoamino)-1-(3-pyridyl)-1-butanone (NNK), which is produced from nicotine through a series of metabolic reactions.^1^^,^^2^ NNK has been shown to cause DNA damage, activate oncogenic signaling pathways, and promote cell proliferation and survival, all of which are hallmarks of cancer development.^1^ Despite extensive research on the molecular mechanism of action of NNK, direct targets that mediate sensitivity to NNK and contribute to its tumorigenicity remain unknown.

Various experimental approaches, including colony formation assays, proliferation studies, and tumor growth in nude mice, have consistently demonstrated NNK facilitation of malignant transformation in human bronchial epithelial cells.^1^ The BEAS-2B cell line is an immortalized but non-tumorigenic line derived from human bronchial epithelium, ideal for understanding this malignant transformation. This cell line has been widely used as a model system to study lung carcinogenesis.^1^ In our study, we comprehensively elucidated the molecular regulatory targets underlying NNK's effect on human lung cancer progression through CRISPR/Cas9 genome-wide knockout screening in BEAS-2B cells. We identified key cellular pathways associated with sensitivity and resistance to NNK, providing novel insights into the molecular mechanisms underlying NNK-induced tumorigenicity and offering potential targets for lung cancer prevention and treatment in smokers.

To gain a comprehensive understanding of potential molecular targets that mediate the sensitivity and resistance to acute and chronic NNK exposure, BRAS-2B cells were transduced with the Brunello CRISPR genome-wide knockout pooled library (Catalog # 73179-LV, 1-vector-system) consisting of 76,411 single-guide RNAs (sgRNAs) targeting 19,114 genes. Building upon the previously established data, we subjected transduced BEAS-2B cells to NNK treatment at a concentration of 100 mg/L for 24 h (Fig. 1A).^1^ Cell viability assays confirmed that NNK accelerates malignant transformation as evidenced by a significantly higher proliferation rate in Brunello-transduced BEAS-2B cells following NNK exposure as compared with the control-treated cells at two time points (1 and 4 weeks) (Fig. 1B). In both NNK-exposed and control lines, proliferation rate increased with time, consistent with an adaptive evolutionary process. Remarkably, after 72 h of NNK treatment, visible colonies were detected in the transduced NNK-exposed cell population (Fig. S1). While maintaining a representation of at least 300 cells per sgRNA in each collection, we isolated gDNA from cells, and sgRNA genes were amplified by PCR and deep-sequenced at an average depth of 31 million reads per sample. After normalizing beta scores by cell cycle-relevant genes, we identified genes that modulate NNK sensitivity and resistance by computational analysis using the MAGeCK pipeline (Fig. S2).

**Figure 1:**
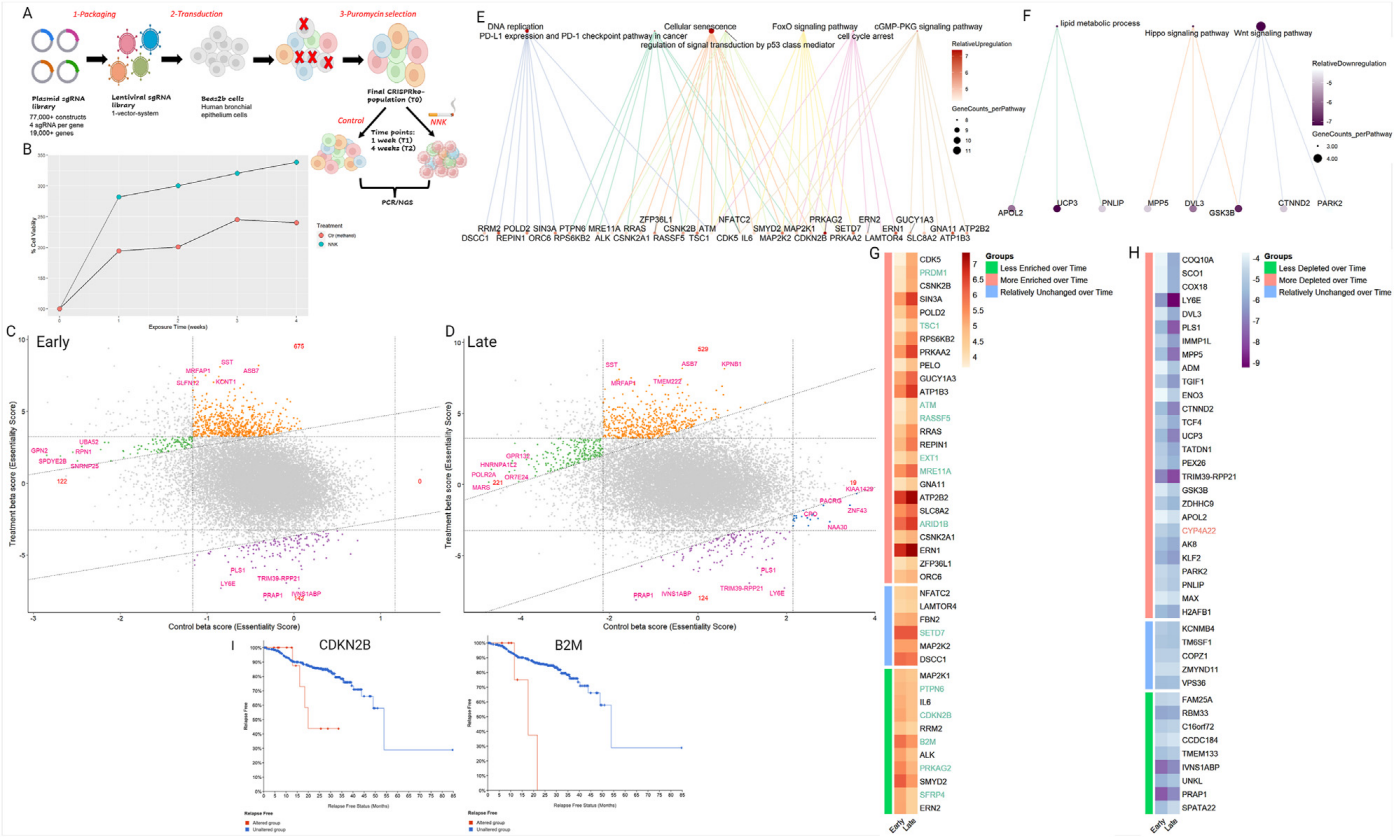
CRISPR/Cas9 genome-wide disruption screening in NNK exposed BEAS-2B cells and bioinformatic analyses. **(A)** Schematic workflow for the genome-wide CRISPR/Cas9 screen. **(B)** The viability of transduced BEAS-2B cells was detected by cell counting during each collection and divided by seeding cell number (t-test, *P* < 0.05). **(C, D)** Nine-square scatterplots for identifying treatment-associated genes. Genes in green and blue groups are found to be strongly negatively or positively selected, respectively, in the control samples, but they are weakly selected in the treatment samples. Genes in the orange group have a strong positive selection in NNK-treated cells as compared with control; these genes are denoted as NNK-resistant genes. Purple-encoded genes are highly negatively selected in the treatment sample; these genes are denoted as NNK-sensitive genes. The numbers in red indicate the number of genes classified in each group. **(E, F)** Functional analysis for treatment-associated genes. Pathway enrichment analysis was done via MAGeCK for commonly enriched **(E)** and commonly depleted **(F)** genes in NNK treatment identified by our CRISPR screening. Top-row dots represent significantly enriched pathways with the most numbers of genes; dot size indicates the number of genes belonging to the noted pathway. Bottom-row dots represent genes that belong to the above significantly enriched pathways; the dot color indicates the differential beta score of the noted gene in NNK treatment versus control/methanol treatment. **(G, H)** Heatmap of the top commonly enriched NNK resistant genes **(G)** and top commonly depleted NNK sensitive genes **(H)** among the identified pathways. Highlighted in green are 13 tumor suppressor genes (TSGs), annotated using the Cancer Gene Census dataset (Genome Version GRCh38 · COSMIC v98). **(I)** Kaplan–Meier survival plot comparing relapse-free survival (RFS) of 466 smoking lung cancer patients with mutated (red line) versus wild-type (blue line) CDKN2B (*P* = 0.0266) or *P* = 2.089e-3 genes. Significance was tested by log-rank test. Deep deletion corresponded to 95.5% of CDKN2B mutations and truncating mutations corresponded to 57.1% of those in B2M. The data were retrieved from the lung adenocarcinoma dataset of the Memorial Sloan Kettering Cancer Center study (MSK, J Thorac Oncol 2020).

To capture the influence of exposure time on NNK-related gene perturbations, we analyzed a nine-square scatterplot for early and late time points independently (Fig. 1C, D). We focused specifically on genes of which perturbations showed increased resistance or sensitivity to NNK treatment. Our analysis revealed 675 genes to be strongly enriched in the NNK sample compared with the control at the early time point (Fig. 1C), while 529 genes showed strong enrichment at the late time point (Fig. 1D). These genes likely play a role in conferring resistance to NNK-induced transformation, as their knockout sensitized BEAS-2B cells to NNK treatment (Fig. S3A). On the flip side, our analysis revealed 142 genes that were significantly depleted at the early time point, along with 124 genes at the late time point, suggesting their essentiality and sensitivity to NNK-induced transformation (Fig. S3B). Further investigation of commonly enriched and depleted genes at the two time points was conducted to gain deeper insights into the molecular mechanisms underlying NNK-mediated effects (Fig. S4A, B). The 404 commonly enriched genes in our screen were also evaluated for overrepresentation of functional pathways using MAGeCK (Fig. 1E). These genes encode cellular components intimately connected to cancer progression, including DNA replication, cancer regulation by PD-L1, cellular senescence, cell cycle arrest, and signal regulation by P53, FoxO, and cGMP-PGK signaling pathways. Thus, activation of these pathways and intact function of the associated genes may enhance DNA repair mechanisms, immune surveillance, growth inhibitory responses, and stress-induced cell fate decisions, collectively reducing the likelihood of NNK-mediated cellular transformation. Annotating the enriched genes in the identified pathways based on the Cancer Gene Census database, we identified 13 hits among these commonly enriched genes as known tumor suppressors (PRDM1, SETD7, ATM, RASSF5, TSC1, EXT1, PTPN1, SFRP4, CDKN2B, B2M, ARID1B, MRE11A, and PRKAG2) (Fig. 1G). Herein, the knockout of cyclin-dependent kinase 2 (CDKN2B) and histone-lysine N-methyltransferase (SETD7), both well-known for their crucial roles in regulating the cell cycle, rendered BEAS-2B cells more vulnerable to the transforming effects of NNK, potentially playing a significant role in protecting against the development of lung cancer upon smoking exposure.^3^^,^^4^ Additionally, the loss of genes such as the serine/threonine kinase ATM (ataxia telangiectasia mutated) and meiotic recombination 11 homolog A (MRE11A) impaired the ability of the cells to properly detect and repair DNA damage caused by NNK. This finding aligns with previous research demonstrating that reduced promoter activity of the MRE11A gene, involved in recognizing DNA damage and activating ATM, increased the risk of lung cancer among smokers.^4^ Interestingly, among our identified relevant tumor suppressors, mutations in two of them (CDKN2B and B2M) displayed a worse survival rate among 466 lung cancer smoking patients across two lung adenocarcinoma studies (Fig. 1I). This observation highlights the clinical relevance of these genes in the context of smoking-related lung cancer. Finally, the robust enrichment of genes associated with cellular senescence highlights its crucial role in countering the harmful effects of NNK and underscores its potential as a target for therapeutic interventions aimed at reducing NNK-induced lung carcinogenesis.

On the other side, the commonly depleted genes encode for pathways involved in Wnt and Hippo signaling as well as lipid metabolism, all of which have been implicated in lung cancer development and tumor initiation and progression (Fig. 1F). In our screen, the knockout of the underlying 79 commonly depleted genes in NNK samples inhibited the NNK transformation in BEAS-2B cells indicating a potential sensitivity for NNK transforming effect of these genes (Fig. 1H). Notably, one of the depleted sgRNA targets in NNK samples was cytochrome P450 family 4 subfamily A member 22 (CYP4A22), a member of the cytochrome P450 (CYP) enzyme family.^1^ Interestingly, CYP4A22 is an orphan CYP with an unknown function in the context of NNK exposure. Knowing that the p450 cytochrome family is responsible for NNK metabolism, further investigation is warranted to elucidate CYP4A22's precise role and mechanisms of action in NNK-induced effects.^5^

Our study is the first to unveil a comprehensive landscape of genetic perturbations and potential targets involved in the sensitivity and resistance to NNK, shedding light on unexplored mechanisms and targets for NNK-induced effects, while also re-affirming that NNK directly interacts with DNA repair and cell cycle control machinery. Further investigations into these identified pathways and genes will deepen our understanding of the molecular events driving NNK-induced transformation and may contribute to the development of novel strategies for intervention, prevention, and personalized treatment of NNK- and smoking-associated lung cancer.

## Conflict of interests

The authors declare that there is no competing interests.

## Funding

This work was supported by grants from the 10.13039/100000002National Institutes of Health (No. R01CA271540, R00CA226506 to CM; No. R01CA196643, R01CA264320, R01CA260629, P50CA150964, and P30 CA043703 to ZW).

## Figures and Tables

**figs1:**
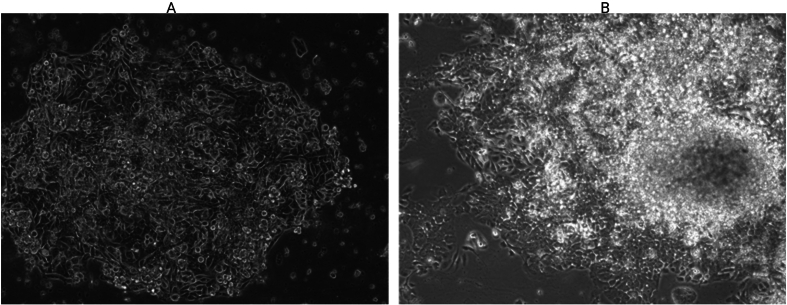


**figs2:**
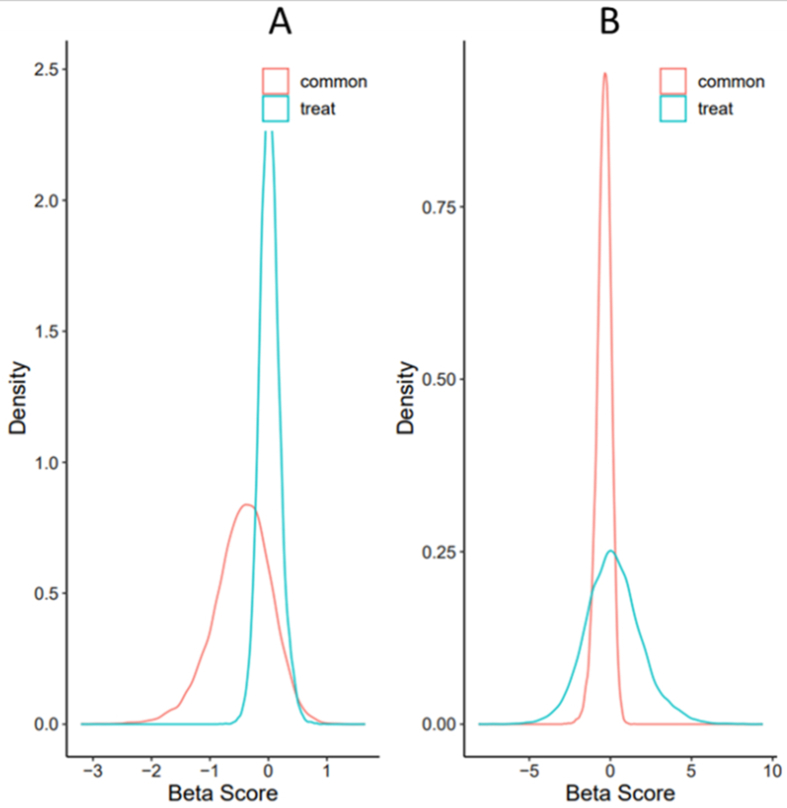


**figs3:**
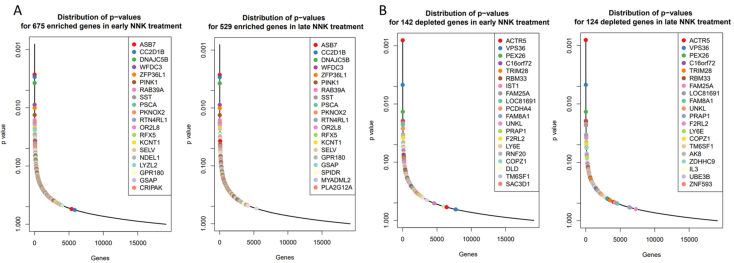


**figs4:**