# Supplementary material for: Exploring the molecular landscape of NNK-induced transformation: A comprehensive genome-wide CRISPR/Cas9 screening
Source: Genes Dis. 2023 Sep 29;11(4):101131. doi: 10.1016/j.gendis.2023.101131 (PMC10915513; doi:10.1016/j.gendis.2023.101131)
Supplement: Multimedia component 1 [file mmc1.docx]

**Supplementary Materials and Methods**

*Cell lines and chemicals*

Human bronchial epithelial Beas-2B cells isolated from normal human lung (cat# CRL-9609) were purchased from the American Type Culture Collection (ATCC, Lnc, U.S.) and cultured in coated plates according to ATCC instructions. Culture flasks were coated with fibronectin, bovine collagen type I and bovine serum albumin dissolved in Bronchial Epithelial Cell Growth Medium (BEGM cat# CC-3170). Bronchial Epithelial Cell Medium (Cat #3211) was used to culture Beas-2B cells consisting of basal medium, bronchial epithelial cell growth supplement (BEpiCGS, Cat. No. 3262), and penicillin/streptomycin solution (P/S, Cat. No. 0503). Cell were kept in an incubator with 5% carbon dioxide (CO2) at 37 °C.

4-(Methylnitrosamino)-1-(3-pyridyl)-1-butanone (NNK) solution was purchased from Sigma Millipore dissolved in methanol at a concentration of 1.0 mg/mL (cat# N-076-1ML). Building upon the previously established data, we subjected transduced BEAS-2B cells to NNK treatment at a concentration of 100 mg/L for 24hrs. Several other valuable studies that have corroborated NNK's ability to transform Beas-2B cells in vitro [1-4].

*Genome-wide CRISPR/Cas9 screen in BEAS-2B cells*

The Brunello CRISPR knockout pooled library, encompassing 76,441 single guide RNAs (sgRNAs) designed to target 19,114 genes within the human genome, was employed to conduct comprehensive whole genome loss-of-function screening. The human Brunello CRISPR knockout pooled library lentiviral prep (Catalog # 73179-LV, 1-vector-system), acquired from Addgene, was used for this purpose. Prior to the screening process, the transduction efficiency of the lentiviral prep was experimentally evaluated in Beas-2B cells. Additionally, a puromycin kill curve was performed to determine the minimum concentration required to eradicate all cells, resulting in the determination of 0.2 μg/mL as the optimal concentration. Beas-2B cells were subsequently transduced with the packaged sgRNA lentivirus library at a multiplicity of infection (MOI) of approximately 0.3, employing 8 μg/mL polybrene (Millipore Sigma, cat#). Sufficient cell quantities were transduced to ensure a representation of at least 300 cells per sgRNA, accounting for the transduction efficiency. Following transduction, the media were refreshed one day later, and puromycin selection was initiated. After seven days of puromycin selection, cells were dissociated and enumerated. Approximately 50 million cells were frozen in liquid nitrogen to serve as the day 0 (control) timepoint for subsequent genomic DNA isolation, while the remaining cells were seeded to commence the screening process, with a minimum representation of 500 cells per sgRNA. The cell culture medium was supplemented with NNK or an equivalent amount of methanol (control) to achieve a final concentration of 100 μg/mL for 24 hours. Weekly cell passaging and cell counts were performed to maintain adequate representation. Cells were counted with trypan blue on each passaging. At the 1st and 4th week timepoints, cells were again frozen in liquid nitrogen to facilitate genomic DNA isolation, ensuring a sufficient representation of 300 cells per sgRNA. Genomic DNA (gDNA) extraction was performed using the Puregene Cell Kit (Qiagen, Cat# 158043) following the manufacturer's instructions. The concentration of isolated gDNA samples was determined using Nanodrop spectrophotometry. Subsequently, the sgRNA regions were amplified and indexed for Illumina sequencing using a one-step PCR method and primers specifically designed for the LentiCRISPRv2-based Brunello libraries, as outlined in the Addgene protocol.

*Bioinformatic analysis of CRISPR screen*

Using MAGeCK MLE, counts of sgRNA sequences were first normalized against the distribution of non-targeting control sgRNAs. Beta scores, representing the gene essentiality, were computed for every gene in NNK treatment group and negative control group (methanol) at early and late time points; Wald p-values were used to determine their statistical significance. To ensure compatibility of beta scores across different condition groups, cell cycle normalization method was conducted with MAGeCKFlute. Normalized beta scores were used to identify treatment-associated genes in both positive and negative selections. Via nine-square scatter plots where gene-specific beta scores (essentiality scores) in the NNK-treated group were plotted against corresponding beta scores in the Methanol-treated group, with the cutoff values of two times standard deviations of the beta score distribution, candidate genes that are highly enriched or depleted in the treatment, but not in the control, were selected for further analyses.

Pathway enrichment analysis was performed with the EnrichAnalyzer function from MAGeCKFlute, using genes that are commonly enriched or depleted across the two time points. Top enriched pathways with the most gene counts, along with their belonging genes, were visualized via R script.

*Survival analysis*

Using cBioPortal for Cancer Genomics, a dataset of 466 lung adenocarcinoma patients indicated as "Ever Smoker" under the smoking history category was analyzed for differences in Relapse Free Survival (RFS) between patients with wild-type and mutant gene of interest. For each gene, significance was indicated by log-rank test p-value, and the Kaplain-Meier plot was visualized by cBioPortal. Data were retrieved from the Lung Adenocarcinoma dataset of the Memorial Sloan Kettering Cancer Center study (MSK, J Thorac Oncol 2020), which is available from the cBioPortal repository, [https://www.cbioportal.org/study/summary?id=luad_mskcc_2020](https://cbioportal-datahub.s3.amazonaws.com/brca_metabric.tar.gz).

References:

1. Zhou J, Xiong R, Zhou J, Guan X, Jiang G, Chen Y, Yang Q. Involvement of m6A regulatory factor IGF2BP1 in malignant transformation of human bronchial epithelial Beas-2B cells induced by tobacco carcinogen NNK. Toxicol Appl Pharmacol. 2022 Feb 1;436:115849. doi: 10.1016/j.taap.2021.115849. Epub 2021 Dec 30. PMID: 34974052.
2. Zhou J, Guan X, Xu E, Zhou J, Xiong R, Yang Q. Chimeric RNA RRM2-C2orf48 plays an oncogenic role in the development of NNK-induced lung cancer. iScience. 2022 Dec 2;26(1):105708. doi: 10.1016/j.isci.2022.105708. PMID: 36570773; PMCID: PMC9771722.
3. Chen E, Zhou J, Xu E, Zhang C, Liu J, Zhou J, Li M, Wu J, Yang Q. A genome-wide screen for differentially methylated long noncoding RNAs identified that lncAC007255.8 is regulated by promoter DNA methylation in Beas-2B cells malignantly transformed by NNK. Toxicol Lett. 2021 Aug 1;346:34-46. doi: 10.1016/j.toxlet.2021.04.013. Epub 2021 Apr 16. PMID: 33872747.
4. Wang H, Li X, Zhao G, Xu L, Wang S, Nie M, Hua C, Shang P, Pan L, Zhao J, Qiao L, Liu K, Hu K, Su J, Cai J, Xie F. Analysis of methyl DNA adducts and metabolites in BEAS-2B cells induced by 4-(methylnitrosamino)-1-(3-pyridyl)-1-butanone. Toxicol Mech Methods. 2019 Sep;29(7):499-510. doi: 10.1080/15376516.2019.1611982. Epub 2019 May 28. PMID: 3105031
5. **(B)**


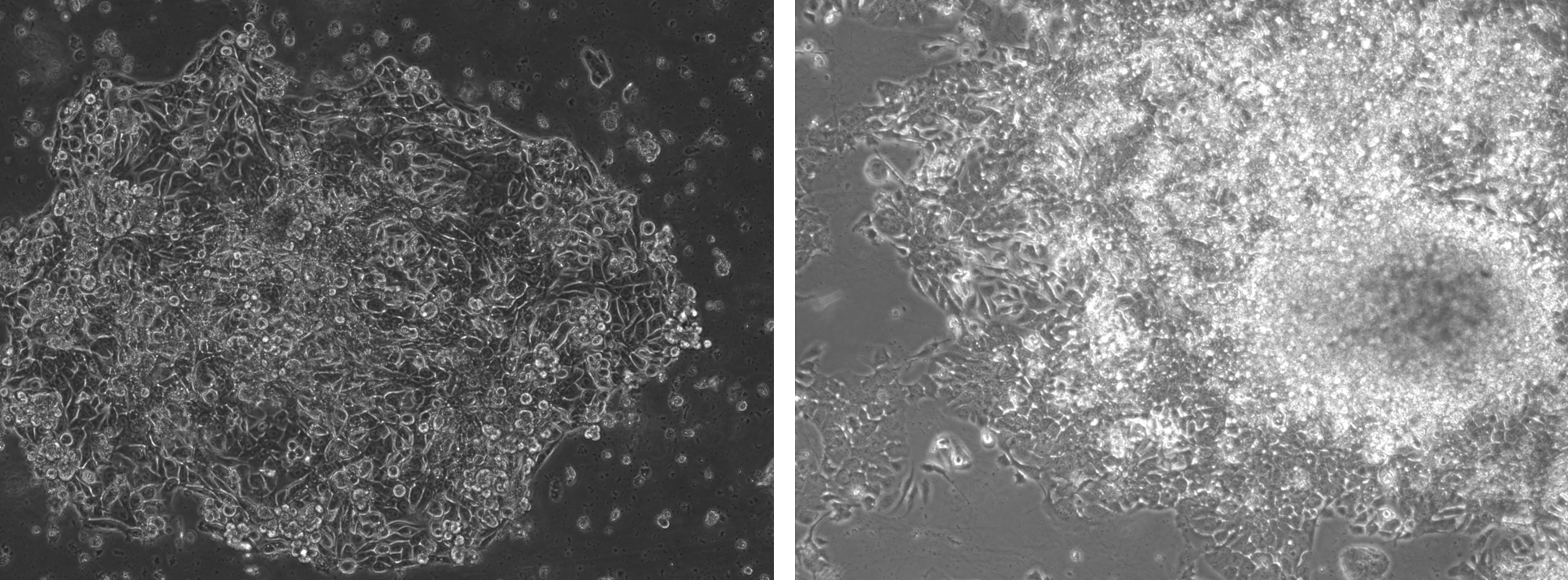


**Supplementary Fig1:** Colony-forming units detected starting 72 hours after treating transduced cells with NNK. Images were captured using light microscope. Magnifications: 10× (A) and 20× (B).


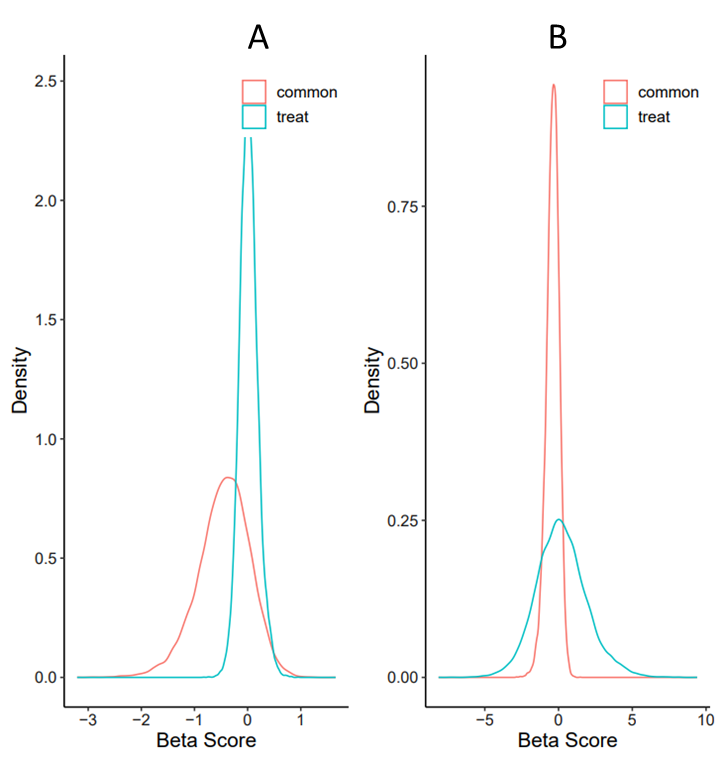


**Supplementary Fig2:** Beta score distributions before (A) and after (B) normalization by the cell cycle normalization method (via MAGeCK) to shorten the gap of the cell cycle in different conditions. Beta score distribution from the 2 conditions had more similar distributions after normalization (B).


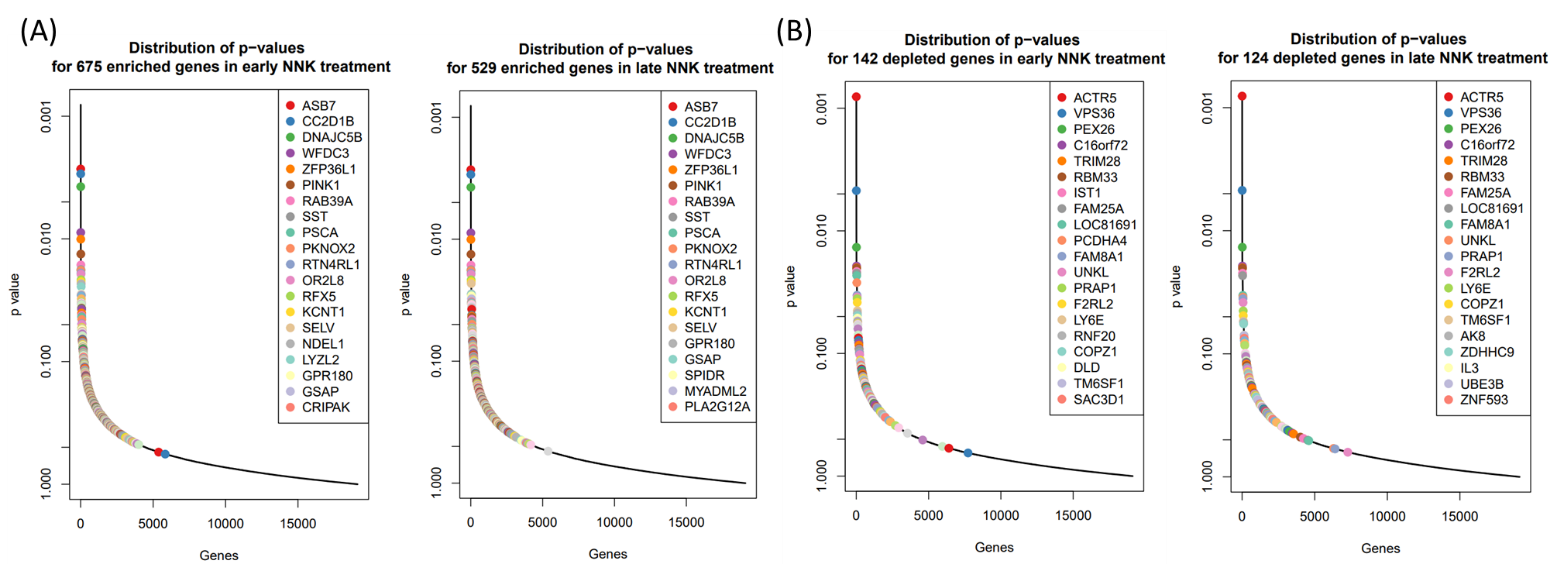


**Supplementary Fig3:** Representative p-value distribution for genes whose sgRNAs are enriched (A) and depleted (B) in NNK treatment at early and late time points. Top 20 genes (according to significant p-values) are listed/labelled in the legends.


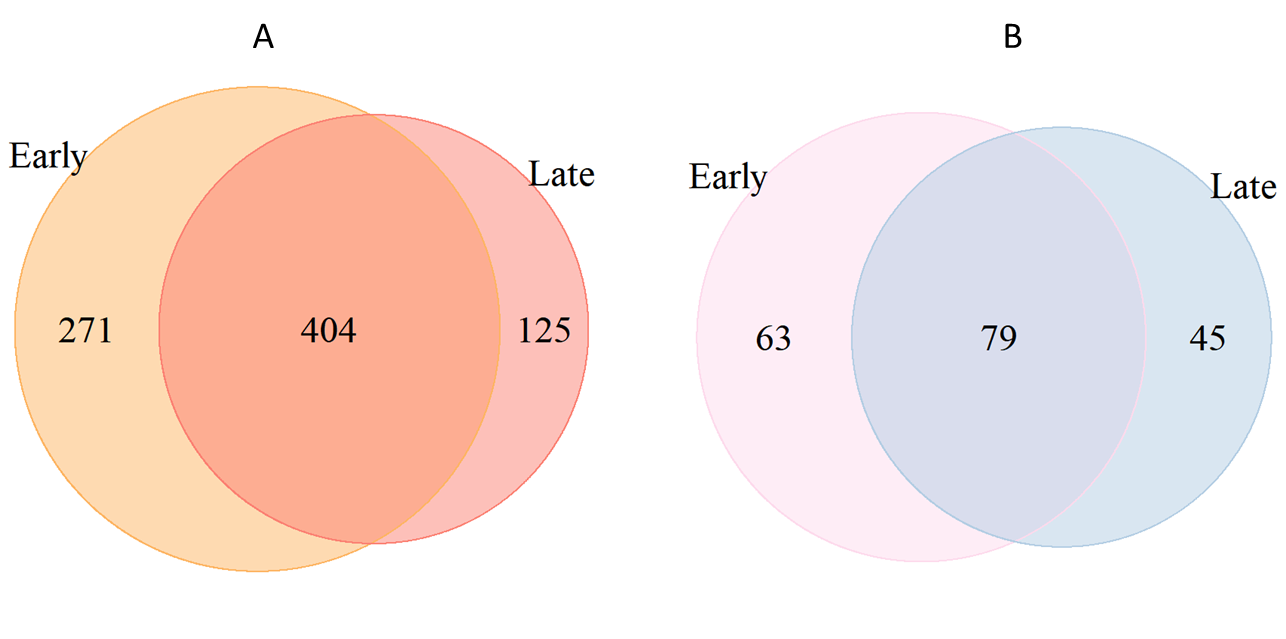


**Supplementary Fig4:** Venn diagrams showing numbers of NNK significant sensitive genes (odd ratio = 20.7 and p-value < 2.2e-16) (A) and NNK resistant genes (odd ratio = 84.7 and p-value < 2.2e-16) (B) at early and late time points. The overlapped sections represent the numbers of genes that are commonly present at both time points.
